# Identificação de Potenciais Biomarcadores Cruciais em IAMCSST por Meio de Análise Bioinformática Integrada

**DOI:** 10.36660/abc.20230462

**Published:** 2024-03-14

**Authors:** Li-Zhi Zhao, Yi Liang, Ting Yin, Hui-Ling Liao, Bo Liang

**Affiliations:** 1 The Affiliated Traditional Chinese Medicine Hospital Southwest Medical University Luzhou China The Affiliated Traditional Chinese Medicine Hospital, Southwest Medical University, Luzhou – China; 2 College of Integration of Traditional Chinese and Western Medicine Southwest Medical University Luzhou China College of Integration of Traditional Chinese and Western Medicine, Southwest Medical University, Luzhou – China; 3 Department of Geriatrics Sichuan Second Hospital of T.C.M. Chengdu China Department of Geriatrics, Sichuan Second Hospital of T.C.M., Chengdu – China; 4 The Second Affiliated Hospital School of Medicine Zhejiang University Hangzhou China Department of Cardiology, The Second Affiliated Hospital, School of Medicine, Zhejiang University, Hangzhou – China; 5 Chongqing Clinical Research Center of Kidney and Urology Diseases Xinqiao Hospital Third Military Medical University Chongqing China Department of Nephrology, The Key Laboratory for the Prevention and Treatment of Chronic Kidney Disease of Chongqing, Chongqing Clinical Research Center of Kidney and Urology Diseases, Xinqiao Hospital, Army Medical University (Third Military Medical University), Chongqing – China

**Keywords:** Infarto do Miocárdio sem Supradesnível do Segmento ST, Doença da Artéria Coronariana, Biomarcadores, Biologia Computacional

## Abstract

**Fundamento:**

O infarto do miocárdio com elevação do segmento ST (IAMCSST) é uma das principais causas de doenças cardiovasculares fatais, que têm sido a principal causa de mortalidade em todo o mundo. O diagnóstico na fase inicial beneficiaria a intervenção clínica e o prognóstico, mas ainda falta a exploração dos biomarcadores do IAMCSST.

**Objetivos:**

Neste estudo, conduzimos uma análise bioinformática para identificar potenciais biomarcadores cruciais no progresso do IAMCSST.

**Métodos:**

Obtivemos GSE59867 para pacientes com IAMCSST e doença arterial coronariana estável (DACE). Genes diferencialmente expressos (GDEs) foram selecionados com o limiar de |log2fold change| > 0,5 e p < 0,05. Com base nesses genes, conduzimos análises de enriquecimento para explorar a relevância potencial entre genes e para rastrear genes centrais. Posteriormente, os genes centrais foram analisados para detectar miRNAs relacionados e DAVID para detectar fatores de transcrição para análise posterior. Finalmente, o GSE62646 foi utilizado para avaliar a especificidade dos GDEs, com genes demonstrando resultados de AUC superiores a 75%, indicando seu potencial como candidatos a biomarcadores. Posteriormente, os genes centrais foram analisados para detectar miRNAs relacionados e DAVID para detectar fatores de transcrição para análise posterior. Finalmente, o GSE62646 foi utilizado para avaliar a especificidade dos GDEs, com genes demonstrando resultados de AUC superiores a 75%, indicando seu potencial como candidatos a biomarcadores.

**Resultados:**

133 GDEs entre DACE e IAMCSST foram obtidos. Em seguida, a rede PPI de GDEs foi construída usando String e Cytoscape, e análises posteriores determinaram genes centrais e 6 complexos moleculares. A análise de enriquecimento funcional dos GDEs sugere que as vias relacionadas à inflamação, metabolismo e imunidade desempenham um papel fundamental na progressão de DACE para IAMCSST. Além disso, foram previstos miRNAs relacionados, has-miR-124, has-miR-130a/b e has-miR-301a/b regularam a expressão do maior número de genes. Enquanto isso, a análise dos fatores de transcrição indica que EVI1, AML1, GATA1 e PPARG são os genes mais enriquecidos. Finalmente, as curvas ROC demonstram que MS4A3, KLRC4, KLRD1, AQP9 e CD14 exibem alta sensibilidade e especificidade na previsão de IAMCSST.

**Conclusões:**

Este estudo revelou que imunidade, metabolismo e inflamação estão envolvidos no desenvolvimento de IAMCSST derivado de DACE, e 6 genes, incluindo MS4A3, KLRC4, KLRD1, AQP9, CD14 e CCR1, poderiam ser empregados como candidatos a biomarcadores para IAMCSST.

**Figura Central:**
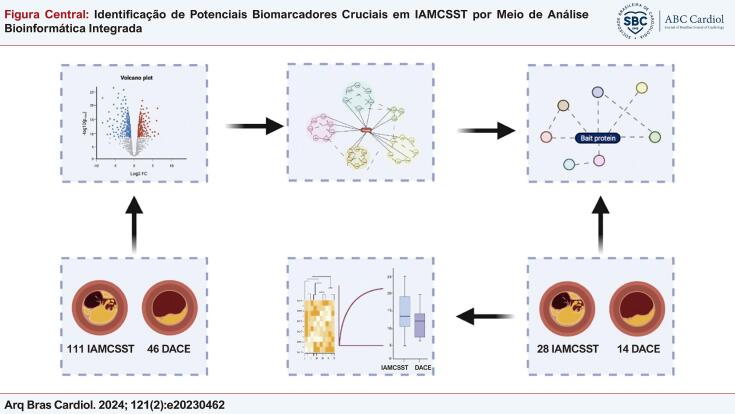
: Identificação de Potenciais Biomarcadores Cruciais em IAMCSST por Meio de Análise Bioinformática Integrada

*Identificação de potenciais biomarcadores cruciais em IAMCSST por meio de análise bioinformática integrada. IAMCSST: infarto do miocárdio com elevação do segmento ST; DACE: doença arterial coronariana estável.*

## Introdução

Nas últimas décadas, as doenças cardiovasculares têm sido a principal causa de mortalidade em todo o mundo.^1^ Entre as mortes por doenças cardiovasculares, a síndrome coronariana aguda (SCA) é a principal causa.^2^ Embora o aumento do uso de estratégias terapêuticas baseadas em evidências e mudanças no estilo de vida tenham estimulado reduções consideráveis na mortalidade por doenças cardiovasculares, o número de mortes ainda está aumentando.^3^ Nos países desenvolvidos, mais de um terço das mortes foram causadas por SCA, e a situação também está a piorar ainda mais nos países em desenvolvimento.^3^

O infarto do miocárdio com elevação do segmento ST (IAMCSST), o tipo mais grave de ataque cardíaco, é um dos três tipos de SCA. Na maioria dos casos, o IAMCSST é devido à ruptura de uma placa aterosclerótica vulnerável num vaso coronário epicárdico, resultando assim numa oclusão trombótica completa,^4^ ou seja, pacientes com doença arterial coronariana estável (DACE) são populações de alto risco de IAMCSST.^3^ Numerosas diretrizes da SCA sugerem que um estilo de vida saudável e um bom desempenho médico reduzem a morbidade, e estratégias de tratamento de reperfusão e revascularização a tempo reduzem a mortalidade.^5 , 6^ No entanto, quando ocorre IAMCSST, é muito difícil detectar, transportar, diagnosticar e realizar operações a tempo, o que dificulta aproveitar o momento de ouro para a revascularização. O diagnóstico de IAMCSST baseia-se na evidência de biomarcadores de necrose miocitária. As isoformas I e T da troponina cardíaca surgiram como os biomarcadores diagnósticos preferidos porque são altamente sensíveis e específicos para lesão miocárdica; portanto, tanto as diretrizes europeias quanto as americanas enfatizam que a troponina cardíaca é o biomarcador preferido para o diagnóstico de IAMCSST.^7 , 8^ Como biomarcadores tradicionais de SCA, a troponina cardíaca e a banda miocárdica da creatina quinase, que seguem cinética semelhante à da troponina cardíaca, têm sido recomendadas para diagnóstico precoce em casos suspeitos de SCA. No entanto, com o desenvolvimento de microarranjos e sequenciamento de última geração, encontrar novos biomarcadores com alta sensibilidade e especificidade é de grande importância para a prevenção e diagnóstico precoce do IAMCSST, especialmente desenvolvido a partir do DACE.

Neste trabalho, investigamos os genes diferencialmente expressos (GDEs) entre pacientes com DACE e IAMCSST. Conduzimos a análise das vias de interação proteína-proteína (PPI), Gene Ontology (GO) e Enciclopédia de Genes e Genomas de Kyoto (KEGG), que ajudaram a elucidar a função dos DEGs. Consequentemente, detectamos microRNAs (miRNAs) e fatores de transcrição relacionados para analisar ainda mais as funções potenciais. Por fim, curvas ROC (Receiver Operating Characteristic) foram traçadas para explorar a sensibilidade e especificidade de potenciais biomarcadores e validar os resultados ( Figura Central ).

## Método

### Aquisição e processamento de dados brutos

Os dados brutos do conjunto de dados de expressão de microarranjo GSE59867^9^ e seu arquivo de anotação GPL6244 foram obtidos do Gene Expression Omnibus. Um total de 157 amostras, incluindo 46 pacientes com DACE sem história de IM e 111 pacientes com IAMCSST, foram incluídos no presente estudo. Os dados são públicos e não envolvem a privacidade dos pacientes, não sendo necessária a revisão e consentimento do comitê de ética.

### Investigação de GDEs

Após o processamento dos dados brutos, analisamos os dados usando o pacote *limma* (versão 3.12) com *fold change* e p para GDEs.^10^ O limite de GDEs foi |log2fold change| > 0,5 e p < 0,05,^11^ e os resultados foram visualizados usando os pacotes *ggplot2* (versão 3.3.3) e *pheatmap* (versão 1.0.12).

### Análise PPI

As informações do PPI foram levantadas utilizando o banco de dados String (versão 11.0).^12^ Em seguida, a rede PPI de GDEs foi carregada no Cytoscape (versão 3.8.2), conforme descrito anteriormente.^13^ OCitoNCA plugin no Cytoscape foi usado para calcular centralidade e avaliar redes biológicas, e o plugin MCODE foi empregado para detectar potenciais complexos moleculares e módulos de função.

### Análise de enriquecimento funcional

Os termos GO e a análise KEGG de GDEs e potenciais complexos moleculares foram realizados usando Metascape, uma plataforma baseada na web que fornece anotação genética, enriquecimento funcional e serviços de análise de interatoma. Termos GO ou vias KEGG com p <0,01 e enriquecidos com mais de 3 genes foram considerados análise de enriquecimento significativo, conforme descrito anteriormente.^14 , 15^

### Análise de Enriquecimento de Conjunto Genético (GSEA)

O GSEA foi conduzido utilizando o software GSEA (versão 7.4) com termos GO, vias KEGG e vias Reactome para complementar o enriquecimento funcional;^16^ termos com p < 0,05 e |escore de enriquecimento normalizado| >1 foram definidos como termos de enriquecimento significativo.

### Investigação de miRNAs essenciais e fatores de transcrição

Investigamos os miRNAs relacionados de genes centrais para uma explicação funcional adicional usando o software FunRich (versão 3.1.4).^17^ Os fatores de transcrição foram previstos usando o Database for Annotation, Visualization, and Integrated Discovery (DAVID, versão 6.8), e os genes enriquecidos classificam os resultados.

### Verificação de genes centrais

Desenhamos as curvas da característica de operação do receptor (ROC) usando o pacote pROC e comparamos a expressão dos genes hub em GSE59867 e GSE62646,^18^ um conjunto de dados genéticos contendo 14 amostras DACE e 28 amostras de IAMCSST, para validar os genes centrais, que poderiam ser os potenciais biomarcadores de IAMCSST. A área sob a curva superior a 75% foi considerada como demonstrando sensibilidade e especificidade excepcionais, o que indicou a sua potencial candidatura como biomarcadores. A expressão dos genes hub foi comparada com um teste t não pareado, e p bicaudal <0,05 foi considerado uma diferença estatística.

## Resultados

### Investigação de GDEs

**Figura 1:**
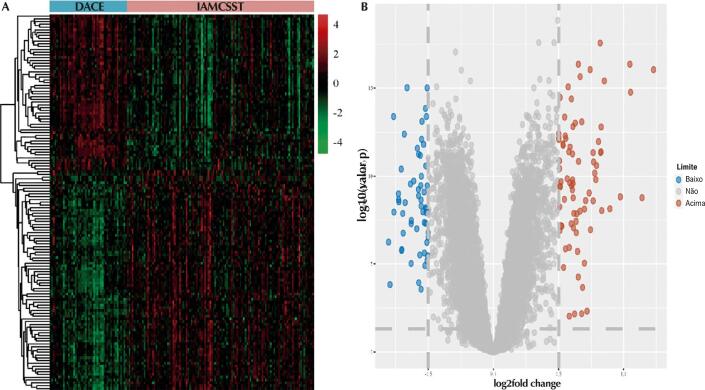
– GDEs em amostras de IAMCSST e amostras de DACE. A) Mapa de calor. Cada linha representa uma amostra e cada coluna representa um único gene. A cor rosa representou amostras de IAMCSST e a cor azul representou amostras DACE. A escala de cores mostra o nível relativo de expressão gênica em determinados slides: verde indica baixos níveis de expressão relativa; vermelho indica altos níveis de expressão relativa. B) Gráfico do vulcão. IAMCSST: infarto do miocárdio com elevação do segmento ST; DACE: doença arterial coronariana estável.

As informações básicas dos conjuntos de dados são mostradas na Tabela 1 . O conjunto de dados de expressão de microarranjo GSE62646 foi avaliado com um boxplot e um gráfico de degradação de RNA; os resultados sugeriram que GSE62646 é um conjunto de dados qualificado ( Figura S1 ). Em seguida, valores incertos ou ambíguos em GSE62646 foram complementados usando o método K-Nearest Neighbor, e os DEGs foram analisados usando o pacote *limma* . Após a regulação pelo UniProt, um total de 133 DEGs foram finalmente determinados, incluindo 54 genes regulados negativamente e 79 genes regulados positivamente, como mostrado na Figura 1 , visualizados pelo gráfico do vulcão e pelo mapa de calor.

**Tabela 1:** – Informações básicas sobre os conjuntos de dados

	GSE59867	GSE62646
Status	Público em 21 de maio de 2015	Público em 23 de outubro de 2014
Título	O perfil de expressão gênica revela potenciais biomarcadores prognósticos associados à progressão da insuficiência cardíaca.	Padrão alterado de expressão gênica em células mononucleares do sangue periférico em pacientes com infarto agudo do miocárdio
População	Um total de 157 amostras, incluindo 111 pacientes com IAMCSST e 46 pacientes com DACE sem histórico de IM.	Um total de 146 amostras, incluindo 28 pacientes com IAMCSST e 14 pacientes com DACE sem histórico de IM.
Tipo de experimento	Perfil de expressão por array	Perfil de expressão por array
Plataformas	GPL6244	GPL6244
Publicação	PMID: 25984239	PMID: 23185530

IAMCSST: infarto do miocárdio com elevação do segmento ST; DACE: doença arterial coronariana estável; IM: infarto do miocárdio.

### Análise PPI

**Figura 2:**
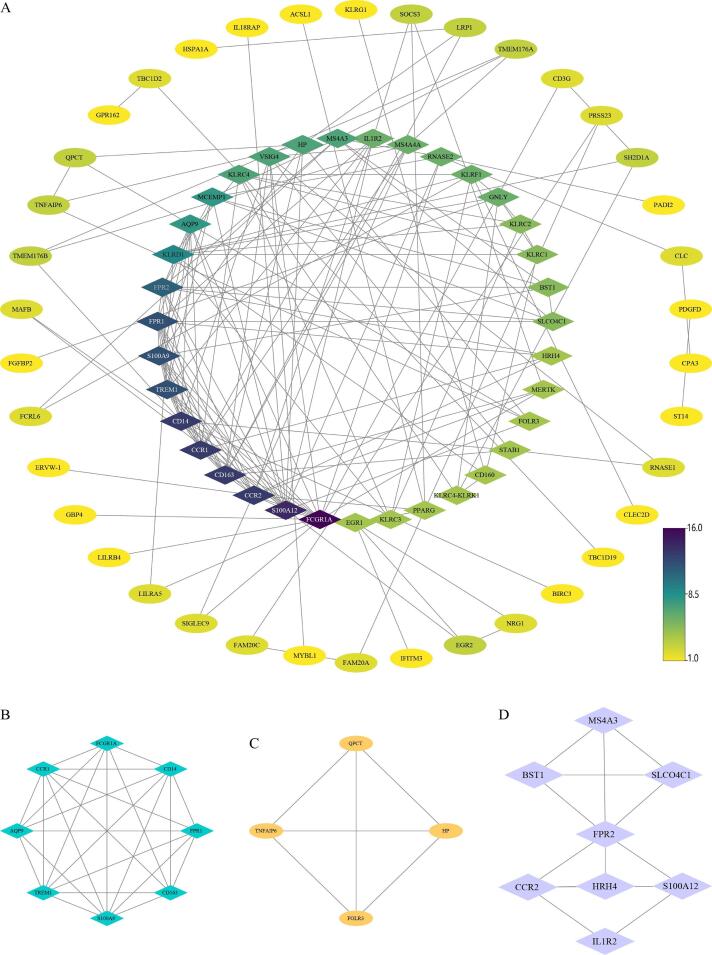
– Rede PPI. A) Toda a rede PPI. B) Módulos biofuncionais M1. C) Módulos biofuncionais M2. D) Módulos biofuncionais M3.

Usando a plataforma String, investigamos a rede PPI de linha e o resultado foi carregado no Cytoscape para processamento posterior. Conforme mostrado na Figura 2A , a rede consistia em 73 nós e 167 arestas; 50 nós desconectados foram ocultados, o valor do grau na rede PPI foi detectado usando CytoNCA para descobrir os genes do hub com a mediana do valor do grau, 35 genes incluindo FCGR1A, S100A12, CD163, CCR2, CD14 e outros foram definidos como genes centrais. Então, o plugin MCODE no Cytoscape foi empregado para detectar os módulos de função potencial ou complexo proteico, como mostrado na Figura 2B ~ D ; os 3 principais módulos de função potencial (M1, M2 e M3) foram selecionados para análise de enriquecimento subsequente.

### Análise de enriquecimento de função

**Figura 3:**
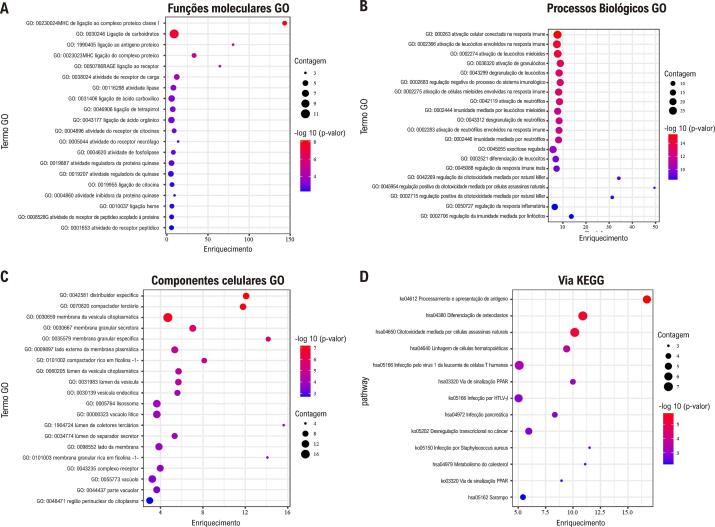
– Análise de enriquecimento GO e KEGG de alvos potenciais. A) Função molecular GO. B) Processos biológicos GO. C) Componentes celulares GO. D) KEGG.

Numerosas funções moleculares estavam envolvidas na ligação do complexo proteico de classe I do MHC, na ligação a carboidratos, na ligação ao antígeno proteico e na ligação ao receptor RAGE ( Figura 3A ). Da mesma forma, numerosos processos biológicos estiveram envolvidos na ativação celular envolvida na resposta imune, ativação de leucócitos envolvida na resposta imune, regulação negativa do processo do sistema imunológico, ativação de células mieloides envolvidas na resposta imune, ativação de neutrófilos, imunidade mediada por leucócitos mieloides e ativação de neutrófilos envolvida na resposta imune ( Figura 3B ). Os resultados indicaram que numerosos componentes celulares estavam envolvidos em grânulos específicos, grânulos terciários, membranas de vesículas citoplasmáticas, membranas granulares secretoras, membranas granulares específicas, o lado externo da membrana plasmática e outros ( Figura 3C ). As vias KEGG estavam envolvidas no processamento e apresentação de antígenos, citotoxicidade mediada por células natural killer, diferenciação de osteoclastos, linhagem de células hematopoiéticas, desregulação transcricional em câncer, infecção pelo vírus 1 da leucemia de células T humanas, infecção por HTLV-I e via de sinalização PPAR ( Figura 3D ). Além disso, a análise funcional dos 3 módulos funcionais potenciais (M1, M2 e M3) esteve envolvida na resposta imune ( Figura S2A~S2C ).

### GSEA

**Figura 4:**
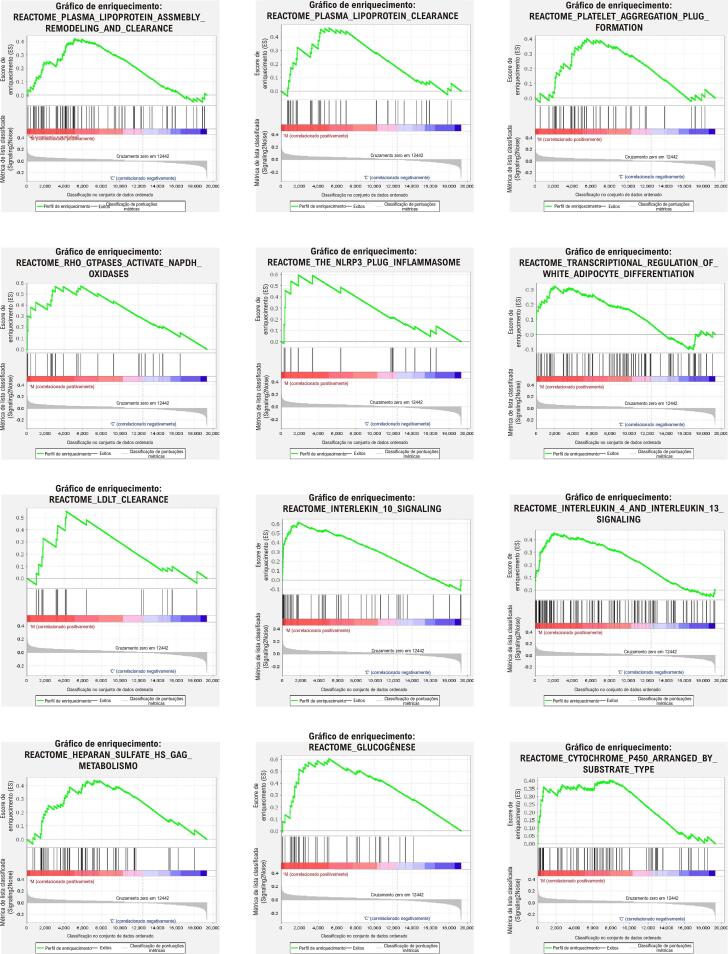
– GSEA.

Para investigar genes que não são expressos de forma significativamente diferencial, mas são biologicamente importantes e para complementar a análise GO e KEGG, foi realizada uma análise GSEA de todo o conjunto de dados usando GSEA. Tomando o ponto de corte mencionado acima, GSEA estava envolvido na remodelação e depuração do conjunto de lipoproteínas plasmáticas, depuração de lipoproteínas plasmáticas, formação de tampão de agregação plaquetária, RHO GTPases ativam NADPH oxidases, inflamassoma NLRP3, regulação transcricional da diferenciação de adipócitos brancos, depuração de LDL, sinalização de interleucina 10, sinalização de interleucina 4 e interleucina 13, metabolismo de sulfato de heparano/heparina, gliconeogênese e citocromo p450 organizados por tipo de substrato ( Figura 4 ). Obviamente, a análise da GSEA enfatizou a importância da resposta imunológica e forneceu suplementos significativos na coagulação e no IAMCSST.

### Previsão adicional de miRNAs e fatores de transcrição

Os miRNAs dos genes centrais foram previstos usando o software FunRich. Os 8 principais miRNAs classificados por grau, incluindo hsa-miR-124, hsa-miR-130a/b, hsa-miR-301a/b, hsa-miR-3666, hsa-miR-4295 e hsa-miR-454 ( Figura S3 ), entre eles foi confirmado que o hsa-miR-124 é fundamental no desenvolvimento de IAMCSST. Enquanto isso, os fatores de transcrição foram analisados utilizando a plataforma DAVID, e os resultados indicam que EVI1, AML1, GATA1 e PPARG são enriquecidos pela maioria dos genes ( Figura S4 ).

### Verificação de genes centrais

**Figura 5:**
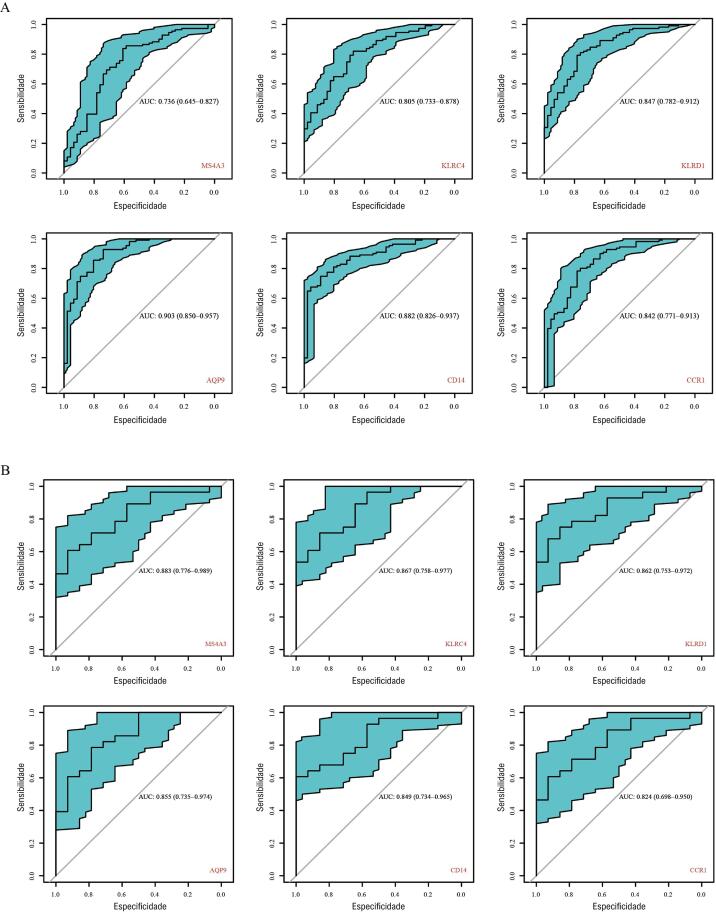
– Curvas ROC de genes centrais. A) GSE59867. B) GSE62646.

**Figura 6:**
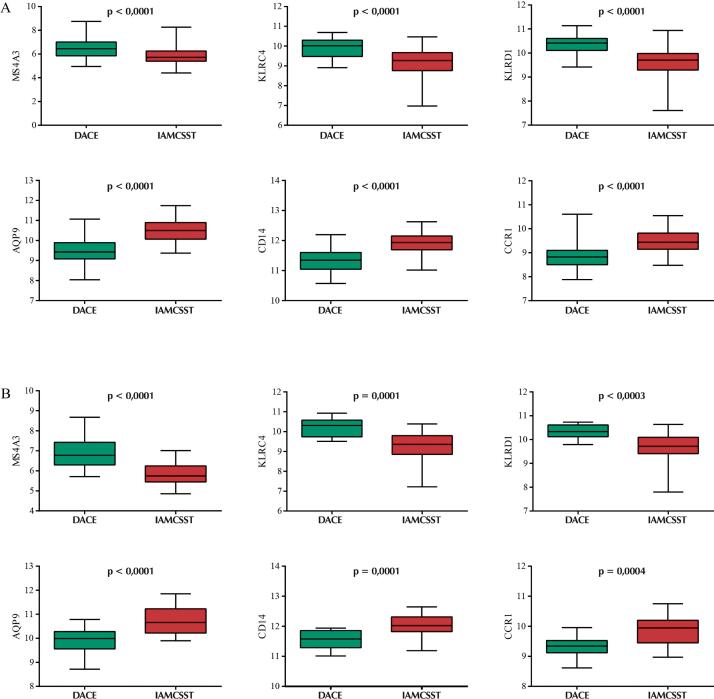
– Expressão diferencial de genes hub. A) GSE59867. B) GSE62646. IAMCSST: infarto do miocárdio com elevação do segmento ST; DACE: doença arterial coronariana estável.

Para detectar a sensibilidade e especificidade dos genes centrais, curvas ROC foram empregadas para a verificação dos genes centrais. Em GSE59867, as AUCs de MS4A3, KLRC4, KLRD1, AQP9, CD14 e CCR1 foram 73,6%, 80,5%, 84,7%, 90,3%, 88,2% e 84,2%, respectivamente (todos p <0,0001) ( Figura 5A , Tabela 2 ), o que indica que esses genes possuem excelente sensibilidade e especificidade. Após processar o conjunto de dados GSE62646, incluindo 14 pacientes com DACE e 28 pacientes com IAMCSST, identificamos que MS4A3, KLRC4, KLRD1, AQP9, CD14 e CCR1 apresentaram sensibilidade e especificidade na predição de IAMCSST ( Figura 5B ). As AUCs desses genes foram de 88,3%, 86,7%, 86,2%, 85,5%, 84,9% e 82,4%, respectivamente (p <0,001) ( Figura 5B , Tabela 2 ). Além disso, MS4A3, KLRC4 e KLRD1 foram regulados negativamente em GSE59867 (todos p <0,0001) ( Figura 6A ) e GSE62646 (p <0,001) ( Figura 6B ), enquanto AQP9, CD14 e CCR1 foram regulados positivamente em GSE59867 (p < 0,0001) ( Figura 6A ) e GSE62646 (p < 0,001) ( Figura 6B ).

**Tabela 2:** – AUCs de genes centrais

	GSE59867				GSE62646			
	AUC	EP	95% IC	p	AUC	EP	95% IC	p
MS4A3	0,736	0,046	0,654~0,827	<0,0001	0,883	0,054	0,776~0,989	<0,0001
KLRC4	0,805	0,037	0,733~0,878	<0,0001	0,967	0,056	0,758~0,977	0,0001
KLRD1	0,847	0,033	0,782~0,912	<0,0001	0,862	0,056	0,753~0,972	0,0002
AQP9	0,903	0,027	0,850~0,957	<0,0001	0,855	0,060	0,735~0,974	0,0002
CD14	0,882	0,028	0,826~0,937	<0,0001	0,849	0,058	0,734~0,965	0,0003
CCR1	0,842	0,036	0,771~0,913	<0,0001	0,824	0,064	0,698~0,950	0,0007

AUC: área sob a curva característica de operação do receptor; EP: erro padrão; IC: intervalo de confiança.

## Discussão

As doenças cardiovasculares são a principal causa de morte no mundo,^1^ e entre eles o IAMCSST deve ser o primeiro a ser controlado. Nos últimos anos, com o rápido desenvolvimento de microarranjos e sequenciamento de próxima geração, é viável e disponível a busca de biomarcadores confiáveis, que beneficiam o diagnóstico precoce e a prevenção do IM. Neste estudo, obtivemos 133 GDEs entre DACE e IAMCSST, então a rede PPI foi construída, e análises posteriores determinaram genes centrais e 6 complexos moleculares. A análise de enriquecimento funcional revelou que a imunidade, o metabolismo e a inflamação estão envolvidos no desenvolvimento do IAMCSST. Além disso, foram previstos 103 miRNAs relacionados, hsa-miR-124, hsa-miR-130a/b e hsa-miR-301a/b regulam o maior número de genes; entretanto, numerosos fatores de transcrição foram investigados, EVI1, AML1, GATA1 e PPARG são enriquecidos pela maioria dos genes. Por fim, as curvas ROC indicam que MS4A3, KLRC4, KLRD1, AQP9 e CD14 possuem alta sensibilidade e especificidade na predição de IAMCSST.

Além disso, as análises GO e KEGG revelaram que a imunidade, o metabolismo e a inflamação estão envolvidos no mecanismo de desenvolvimento de IAMCSST. Como mostrado acima, vários termos de enriquecimento estavam envolvidos na imunidade e na inflamação, incluindo ativação celular envolvida na resposta imune, ativação de leucócitos envolvida na resposta imune, regulação negativa do processo do sistema imunológico, regulação da imunidade mediada por células assassinas naturais e assim por diante. Enquanto guardiões do sistema imunológico, os leucócitos possuem regulação bidirecional para o desenvolvimento de IAMCSST; alguns leucócitos são aterogênicos, enquanto outros são ateroprotetores; alguns sustentam a inflamação após o infarto do miocárdio, enquanto outros a resolvem.^19^ Uma revisão construiu um modelo das estratégias terapêuticas do IAMCSST, estudos experimentais anteriores revelaram mecanismos complexos em relação ao desenvolvimento, reparação e remodelação do IAMCSST e modulação da inflamação individualmente com base nas características da condição do paciente no benefício dos pacientes com IAMCSST.^20^ Resultados semelhantes puderam ser encontrados na análise funcional do potencial complexo molecular; todos os 3 complexos moleculares potenciais estavam envolvidos na imunidade. Como complemento à análise GO e KEGG, a análise GSEA verificou os resultados da análise funcional e forneceu mais evidências de metabolismo. Além dos resultados da análise de enriquecimento sugerindo que os DEGs estão relacionados ao metabolismo do colesterol, os resultados do GSEA levantaram vários termos envolvidos na regulação do metabolismo do colesterol, incluindo remodelação e depuração do conjunto de lipoproteínas plasmáticas, depuração de lipoproteínas plasmáticas, depuração de LDL e sulfato de heparano. /metabolismo da heparina, que eram consistentes com a cognição atual. Além disso, os resultados da análise GSEA indicaram que a regulação da formação do tampão de agregação plaquetária foi diferente entre DACE e IAMCSST, enfatizando que a coagulação também é fundamental para o desenvolvimento de IAMCSST.^21^ Além disso, a GSEA destacou a inflamação, o stress oxidativo e o metabolismo dos medicamentos, o que pôde ser confirmado na investigação existente.^22 - 24^ Os miRNAs desempenham papéis fundamentais na gênese e progressão do IAMCSST; após a triagem, hsa-miR-124, hsa-miR-130a/b, hsa-miR-301a/b, hsa-miR-3666, hsa-miR-4295 e hsa-miR-454 foram identificados como os principais miRNAs enriquecidos, numerosos estudos ilustram que o hsa-miR-124 regula o estresse oxidativo e a hipóxia no desenvolvimento do infarto do miocárdio, e pode ser um potencial biomarcador, bem como o alvo terapêutico para o IAMCSST.^25 , 26^ Na família MiR-130, incluindo miR-130a e miR- 130b, uma análise mostra que o miR-130 agrava o IAMCSST ao atingir a via PPAR-γ.^27^ São necessárias pesquisas para explorar e validar a conexão entre hsa-miR-301a/b, hsa-miR-3666, hsa-miR-4295 e hsa-miR-454 e IM, além de miRNAs apoiados por experimentos envolvidos no desenvolvimento de IM , como miR-19, miR-23 e outros, podem ser encontrados nos resultados da predição.^28 - 30^ Os resultados da previsão do fator de transcrição filtraram 4 fatores de transcrição, incluindo EVI1, AML1, GATA1 e PPARG. EVI1, histona-lisina N-metiltransferase MECOM, está envolvido no progresso da imunidade, metabolismo e inflamação.^31 , 32^ Da mesma forma, AML1, fator de transcrição 1 relacionado ao runt, está envolvido na regulação funcional da leucemia, células B e células T e regula o sistema imunológico.^33^ O GATA1, também conhecido como fator de transcrição eritróide, está envolvido no progresso da produção e coagulação de plaquetas.^34^ Enquanto isso, um estudo elucidou que o GATA1 está relacionado a uma doença vascular familiar com características de DACE e IAMCSST.^35^ O PPARG, conhecido como receptor gama ativado por proliferador de peroxissoma, é o receptor nuclear que se liga aos proliferadores de peroxissoma, como drogas hipolipemiantes e ácidos graxos, principalmente envolvido no progresso do metabolismo da gordura e da inflamação e é fundamental para o desenvolvimento de IAMCSST.^36^

MS4A3 regula o nível de fosforilação de CDK2 através de sua ligação direta a CDKN3,^37^ e um estudo de coorte sugere que CDK2 estava envolvido na proliferação anormal, uma das características da aterosclerose e do STEMI.^38^ Além disso, um estudo mencionou que o CDK2 está envolvido na regulação do ciclo celular em miócitos após infarto do miocárdio, o que promove a regeneração da massa muscular e a recuperação da função ventricular.^39^ Tanto o KLRD1 quanto o KLRC4 são receptores naturais de células assassinas, e naturais as células killer são importantes no aparecimento do IAMCSST pela sua capacidade de secretar IFN-γ e outras citocinas inflamatórias.^40^ Os pesquisadores mencionaram que a superexpressão do miR-212 inibiu a AQP9 ativando a via de sinalização PI3K/Akt, diminuindo assim a apoptose dos cardiomiócitos, promovendo a regeneração vascular e aliviando a remodelação ventricular em ratos com IAMCSST.^41^ Da mesma forma, um estudo indicou que o silenciamento do gene AQP9 pode inibir a ativação da via de sinalização ERK1/2, atenuar a resposta inflamatória em ratos com IAMCSST, inibir a apoptose de células miocárdicas e melhorar a função cardíaca.^42^ CD14, o nome completo da proteína é antígeno de diferenciação de monócitos CD14, recentemente, um estudo mencionou que, em comparação com pacientes com DAC, os níveis de monócitos relacionados ao CD14 foram significativamente maiores em pacientes com IAMCSST.^43^

Este estudo tem algumas limitações. Primeiro, todos os resultados da análise foram derivados de conjuntos de dados anteriores. Apesar dos esforços que temos feito no controle de qualidade, a autenticidade dos resultados ainda necessita de verificação. Além disso, limitado pelas informações contidas em GSE59867 e GSE62646, não podemos comparar o desempenho diagnóstico dos biomarcadores identificados com a troponina I e T nem avaliar diferenças em sua cinética temporal. Terceiro, todos os dados que utilizamos vieram de células mononucleares do sangue periférico, e não da artéria coronária ou do tecido cardíaco, porque é relativamente difícil obter clinicamente a artéria coronária e o tecido cardíaco. Felizmente, estudos anteriores demonstraram que os dados do sangue periférico também têm boa fiabilidade.^44 , 45^ Finalmente, embora numerosos estudos apoiassem a potencialidade dos potenciais biomarcadores previstos neste estudo, os resultados das curvas ROC não conseguiram encontrar um gene com elevada confiança (AUC > 90%), e são necessários ensaios consideráveis para validar a sensibilidade e especificidade de potenciais biomarcadores. No entanto, este estudo determinou os potenciais biomarcadores e investigou os mecanismos complexos do IAMCSST desenvolvidos a partir da DACE, o que promoveu a designação do nosso próximo plano para explorar os mecanismos no ensaio clínico em breve.

## Conclusão

Revelamos que imunidade, metabolismo e inflamação estão envolvidos no desenvolvimento de IAMCSST derivado de DACE, e 5 genes, incluindo MS4A3, KLRC4, KLRD1, AQP9 e CD14, poderiam ser empregados como candidatos a biomarcadores para IAMCSST.
