# Supplementary material for: Identificação de Potenciais Biomarcadores Cruciais em IAMCSST por Meio de Análise Bioinformática Integrada
Source: Arq Bras Cardiol. 2024 Mar 14;121(2):e20230462. [Article in Portuguese] doi: 10.36660/abc.20230462 (PMC12094287; doi:10.36660/abc.20230462)
Supplement: Translation

# Translation: Identification of Potential Crucial Biomarkers in STEMI Through Integrated Bioinformatic Analysis

**Authors:** Li-Zhi Zhao, Yi Liang, Ting Yin, Hui-Ling Liao, Bo Liang

**Affiliations:** 1 China The Affiliated Traditional Chinese Medicine Hospital, Southwest Medical University, Luzhou – China; 2 China School of Integration of Traditional Chinese and Western Medicine, Southwest Medical University, Luzhou – China; 3 China Department of Geriatrics, Sichuan Second Hospital of T.C.M., Chengdu – China; 4 China Department of Cardiology, The Second Affiliated Hospital, School of Medicine, Zhejiang University, Hangzhou – China; 5 China Department of Nephrology, The Key Laboratory for the Prevention and Treatment of Chronic Kidney Disease of Chongqing, Chongqing Clinical Research Center of Kidney and Urology Diseases, Xinqiao Hospital, Army Medical University (Third Military Medical University),Chongqing – China

**Keywords:** ST Elevation Myocardial Infarction, Coronary Artery Disease, Biomarkers, Computational Biology

## Abstract

**Background:**

ST-segment elevation myocardial infarction (STEMI) is one of the leading causes of fatal cardiovascular diseases, which have been the prime cause of mortality worldwide. Diagnosis in the early phase would benefit clinical intervention and prognosis, but the exploration of the biomarkers of STEMI is still lacking.

**Objectives:**

In this study, we conducted a bioinformatics analysis to identify potential crucial biomarkers in the progress of STEMI.

**Methods:**

We obtained GSE59867 for STEMI and stable coronary artery disease (SCAD) patients. Differentially expressed genes (DEGs) were screened with the threshold of |log2fold change| > 0.5 and p <0.05. Based on these genes, we conducted enrichment analysis to explore the potential relevance between genes and to screen hub genes. Subsequently, hub genes were analyzed to detect related miRNAs and DAVID to detect transcription factors for further analysis. Finally, GSE62646 was utilized to assess DEGs specificity, with genes demonstrating AUC results exceeding 75%, indicating their potential as candidate biomarkers.

**Results:**

133 DEGs between SCAD and STEMI were obtained. Then, the PPI network of DEGs was constructed using String and Cytoscape, and further analysis determined hub genes and 6 molecular complexes. Functional enrichment analysis of the DEGs suggests that pathways related to inflammation, metabolism, and immunity play a pivotal role in the progression from SCAD to STEMI. Besides, related-miRNAs were predicted, has-miR-124, has-miR-130a/b, and has-miR-301a/b regulated the expression of the largest number of genes. Meanwhile, Transcription factors analysis indicate that EVI1, AML1, GATA1, and PPARG are the most enriched gene. Finally, ROC curves demonstrate that MS4A3, KLRC4, KLRD1, AQP9, and CD14 exhibit both high sensitivity and specificity in predicting STEMI.

**Conclusions:**

This study revealed that immunity, metabolism, and inflammation are involved in the development of STEMI derived from SCAD, and 6 genes, including MS4A3, KLRC4, KLRD1, AQP9, CD14, and CCR1, could be employed as candidate biomarkers to STEMI.

**Central Illustration:** : Identification of Potential Crucial Biomarkers in STEMI Through Integrated Bioinformatic Analysis

*Identification of Potential Crucial Biomarkers in STEMI Through Integrated Bioinformatic Analysis. STEMI: ST-segment elevation myocardial infarction; SCAD: stable coronary artery disease.*

## Introduction

In the past decades, cardiovascular diseases have been the prime cause of mortality worldwid.^1^Among the deaths from cardiovascular diseases, acute coronary syndrome (ACS) is the leading cause.^2^Although increased use of evidence-based therapy strategies and lifestyle changes have spurred considerable reductions in mortality from cardiovascular diseases, the number of deaths is still increasing.^3^In developed nations, more than a third of deaths were caused by ACS, and the situation is also rising even worse in developing countries.^3^

ST-segment elevation myocardial infarction (STEMI), the most severe type of heart attack, is one of three types of ACS. In the majority of cases, STEMI is due to disruption of a vulnerable atherosclerotic plaque in an epicardial coronary vessel, thereby, a complete thrombotic occlusion,^4^that is to say, stable coronary artery disease (SCAD) patients are high-risk populations of STEMI,^3^Numerous guidelines from ACS suggest that a healthy lifestyle and good medical performance reduce morbidity and reperfusion and revascularization treatment strategies in time reduce mortality.^5 , 6^Nevertheless, when STEMI occurs, it is very hard to detect, transport, diagnose, and perform operations in time, which makes it difficult to seize the golden time for revascularization. The diagnosis of STEMI relies on biomarker evidence of myocyte necrosis. Cardiac troponin isoforms I and T have emerged as the preferred diagnostic biomarkers because they are highly sensitive and specific for myocardial injury; therefore, both European and American guidelines emphasize that cardiac troponin is the preferred biomarker for diagnosis of STEMI.^7 , 8^As traditional biomarkers of ACS, cardiac troponin and creatine kinase myocardial band, which follow similar kinetics as cardiac troponin, have been recommended for early diagnosis in suspected cases of ACS. However, with the development of microarray and next-generation sequencing, finding new biomarkers with high sensitivity and specificity is of great significance for the prevention and early diagnosis of STEMI, specially developed from SCAD.

In this work, we investigated the differentially expressed genes (DEGs) between patients with SCAD and STEMI. We conducted the protein-protein interaction (PPI), Gene Ontology (GO), and Kyoto Encyclopedia of Genes and Genomes (KEGG) pathway analysis, which helped to elucidate the function of DEGs. Consequently, we detected related microRNAs (miRNAs) and transcription factors to analyze the potential functions further. At last, receiver operating characteristic (ROC) curves were plotted to explore the sensitivity and specificity of potential biomarkers and validate the results ( Central Illustration ).

## Method

### Acquisition and processing of raw data

The raw data of the microarray expression dataset GSE59867^9^and its annotation file GPL6244 were obtained from Gene Expression Omnibus. A total of 157 samples, including 46 SCAD patients without a history of MI and 111 ST-segment elevation myocardial infarction (STEMI) patients, were included in the present study. The data are public and do not involve the privacy of patients, so the review and consent of the ethics committee are not required.

### Investigation of DEGs

After the processing of raw data, we analyzed the data using the *limma* package (version 3.12) with a fold change and p for DEGs.^10^The threshold of DEGs was |log_2_fold change| > 0.5 and p <0.05,^11^and the results were visualized using the *ggplot2* (version 3.3.3) and the *pheatmap* (version 1.0.12) packages.

### PPI analysis

PPI information was surveyed using the String database (version 11.0).^12^Next, the PPI network of DEGs was uploaded to Cytoscape (version 3.8.2), as described previously.^13^The CytoNCA plugin in Cytoscape was used to calculate centrality and evaluate biological networks, and the MCODE plugin was employed for detecting potential molecular complexes and function modules.

### Functional enrichment analysis

GO terms and KEGG analysis of DEGs and potential molecular complexes were carried out using Metascape, a web-based platform providing gene annotation, functional enrichment, and interactome analysis services. GO terms or KEGG pathways with both p < 0.01 and enriched with more than 3 genes were considered significant enrichment analysis, as described previously.^14 , 15^

### Gene Set Enrichment Analysis (GSEA)

GSEA was conducted using GSEA software (version 7.4) with GO terms, KEGG pathways, and Reactome pathways to supplement the functional enrichment;^16^terms with p < 0.05 and |normalized enrichment score| >1 were defined as significant enrichment terms.

### Investigation of pivotal miRNAs and transcription factors

We investigated the related miRNAs of hub genes for a further functional explanation using FunRich software (version 3.1.4).^17^The transcription factors were predicted using the Database for Annotation, Visualization, and Integrated Discovery (DAVID, version 6.8), and the enriched genes rank the results.

### Verification of hub genes

We draw the receiver operating characteristic (ROC) curves using the *pROC* package and compared the expression of hub genes in both GSE59867 and GSE62646,^18^a gene dataset containing 14 SCAD samples and 28 STEMI samples, to validate the hub genes, which could be the potential biomarkers of STEMI. The area under the curve exceeding 75% was regarded as demonstrating exceptional sensitivity and specificity, which indicated their potential candidacy as biomarkers. The expression of hub genes was compared with an unpaired t-test, and two-tailed p < 0.05 was considered a statistical difference.

## Results

### Investigation of DEGs

The basic information of the datasets is shown in Table 1 . Microarray expression dataset GSE62646 was evaluated with a boxplot and RNA degradation plot; the results suggested that GSE62646 is a qualified dataset ( Figure S1 ). Then, uncertain or ambiguous values in GSE62646 were supplemented using the K-Nearest Neighbor method, and the DEGs were analyzed using the *limma* package. After regulating by UniProt, a total of 133 DEGs were finally determined, including 54 downregulated genes, and 79 upregulated genes, as shown in Figure 1 , visualized by volcano plot and heat map.

**Table 1:** – Basic information about the datasets

	GSE59867	GSE62646
Status	Public on May 21, 2015	Public on Oct 23, 2014
Title	Gene expression profiling reveals potential prognostic biomarkers associated with the progression of heart failure.	Altered gene expression pattern in peripheral blood mononuclear cells in patients with acute myocardial infarction
Population	A total of 157 samples, including 111 STEMI patients and 46 SCAD patients without a history of MI.	A total of 146 samples, including 28 STEMI patients and 14 SCAD patients without a history of MI.
Experiment type	Expression profiling by array	Expression profiling by array
Platforms	GPL6244	GPL6244
Publication	PMID: 25984239	PMID: 23185530

STEMI: ST-segment elevation myocardial infarction; SCAD: stable coronary artery disease; MI: myocardial infarction.

**Figure 1:** – DEGs in STEMI samples and SCAD samples. A) Heat map. Each row represents a sample, and each column represents a single gene. Pink color represented STEMI samples, and blue color represented SCAD samples. The color scale shows the relative gene expression level in certain slides: green indicates low relative expression levels; red indicates high relative expression levels. B) Volcano plot. STEMI: ST-segment elevation myocardial infarction; SCAD: stable coronary artery disease.

### PPI analysis

Using the String platform, we investigated the row PPI network, and the result was uploaded to Cytoscape for further processing. As shown in Figure 2A , the network consisted of 73 nodes and 167 edges; 50 disconnected nodes were hidden, the value of the degree in the PPI network was detected using CytoNCA to figure out the hub genes with the median of the degree value, 35 genes including FCGR1A, S100A12, CD163, CCR2, CD14, and others were defined as hub genes. Then, the MCODE plugin in Cytoscape was employed to detect the potential function modules or protein complex, as shown in Figure 2B~D ; the top 3 potential function modules (M1, M2, and M3) were selected for subsequent enrichment analysis.

**Figure 2:** – PPI network. A) The whole PPI network. B) M1 bio-functional modules. C) M2 bio-functional modules. D) M3 bio-functional modules.

### Function enrichment analysis

Numerous molecular functions were involved in MHC class I protein complex binding, carbohydrate binding, protein antigen binding, and RAGE receptor binding ( Figure 3A ). Similarly, numerous biological processes were involved in cell activation involved in the immune response, leukocyte activation involved in the immune response, negative regulation of the immune system process, myeloid cell activation involved in the immune response, neutrophil activation, myeloid leukocyte mediated immunity, and neutrophil activation involved in immune response ( Figure 3B ). The results indicated that numerous cellular components were involved in specific granules, tertiary granules, cytoplasmic vesicle membranes, secretory granule membranes, specific granule membranes, the external side of the plasma membrane, and others ( Figure 3C ). KEGG pathways were involved in Antigen processing and presentation, natural killer cell-mediated cytotoxicity, osteoclast differentiation, hematopoietic cell lineage, transcriptional misregulation in cancer, Human T-cell leukemia virus 1 infection, HTLV-I infection, and PPAR signaling pathway ( Figure 3D ). Besides, the functional analysis of the 3 potential function modules (M1, M2, and M3) was involved in the immune response ( Figure S2A~S2C ).

**Figure 3:** – GO and KEGG enrichment analysis of potential targets. A) GO molecular function. B) GO biological processes. C) GO cellular components. D) KEGG.

### GSEA

To investigate genes that are not significantly differentially expressed but are biologically important and to supplement GO and KEGG analysis, a GSEA analysis of the whole dataset was conducted using GSEA. Take the cutoff as mentioned above, GSEA was involved in plasma lipoprotein assembly remodeling and clearance, plasma lipoprotein clearance, platelet aggregation plug formation, RHO GTPases activate NADPH oxidases, NLRP3 inflammasome, transcriptional regulation of white adipocyte differentiation, LDL clearance, interleukin 10 signaling, interleukin 4 and interleukin 13 signaling, heparan sulfate/heparin metabolism, gluconeogenesis, and cytochrome p450 arranged by substrate type ( Figure 4 ). Obviously, GSEA analysis emphasized the importance of immune-related response and has provided significant supplements on coagulation and STEMI.

**Figure 4:** – GSEA.

### Further prediction of miRNAs and transcription factors

The miRNAs of hub genes were predicted using FunRich software. Top 8 miRNA ranked by degree, including hsa-miR-124, hsa-miR-130a/b, hsa-miR-301a/b, hsa-miR-3666, hsa-miR-4295, and hsa-miR-454 ( Figure S3 ), among them hsa-miR-124 was confirmed are pivotal in the development of STEMI. Meanwhile, transcription factors were analyzed using the DAVID platform, and the results indicate that EVI1, AML1, GATA1, and PPARG are enriched by most genes ( Figure S4 ).

### Verification of hub genes

To detect the sensitivity and specificity of hub genes, ROC curves were employed for the verification of the hub genes. In GSE59867, the AUCs of MS4A3, KLRC4, KLRD1, AQP9, CD14, and CCR1 were 73.6%, 80.5%, 84.7%, 90.3%, 88.2%, and 84.2%, respectively (all *P* < 0.0001) ( Figure 5A , Table 2 ), which indicate that these genes have excellent sensitivity and specificity. After processing the GSE62646 dataset, including 14 SCAD patients and 28 STEMI patients, we identified that MS4A3, KLRC4, KLRD1, AQP9, CD14, and CCR1 had sensitivity and specificity in the prediction of STEMI ( Figure 5B ). The AUCs of these genes were 88.3%, 86.7%, 86.2%, 85.5%, 84.9%, and 82.4%, respectively (p < 0.001) ( Figure 5B , Table 2 ). In addition, MS4A3, KLRC4, and KLRD1 were down-regulated in GSE59867 (p < 0.0001) ( Figure 6A ) and GSE62646 (p < 0.001) ( Figure 6B ), whereas AQP9, CD14, and CCR1 were upregulated in GSE59867 (p < 0.0001) ( Figure 6A ) and GSE62646 (p < 0.001) ( Figure 6B ).

**Figure 5:** – ROC curves of hub genes. A) GSE59867. B) GSE62646.

**Table 2:** – AUCs of hub genes

	GSE59867				GSE62646			
	AUC	SE	95% CIs	p	AUC	SE	95% CIs	p
MS4A3	0.736	0.046	0.654~0.827	< 0.0001	0.883	0.054	0.776~0.989	< 0.0001
KLRC4	0.805	0.037	0.733~0.878	< 0.0001	0.967	0.056	0.758~0.977	0.0001
KLRD1	0.847	0.033	0.782~0.912	< 0.0001	0.862	0.056	0.753~0.972	0.0002
AQP9	0.903	0.027	0.850~0.957	< 0.0001	0.855	0.060	0.735~0.974	0.0002
CD14	0.882	0.028	0.826~0.937	< 0.0001	0.849	0.058	0.734~0.965	0.0003
CCR1	0.842	0.036	0.771~0.913	< 0.0001	0.824	0.064	0.698~0.950	0.0007

AUC: area under the receiver operating characteristic curve; SE: standard error; CI: confidence interval.

**Figure 6:** – Differential expression of hub genes. A) GSE59867. B) GSE62646. STEMI: ST-segment elevation myocardial infarction; SCAD: stable coronary artery disease.

## Discussion

Cardiovascular diseases are the leading cause of death worldwide,^1^and among them, STEMI should be the first to be controlled. In recent years, with the rapid development of microarray and next-generation sequencing, it is feasible and available to seek reliable biomarkers, which benefit the early diagnosis and prevention of MI. In this study, we obtained 133 DEGs between SCAD and STEMI, then the PPI network was constructed, and further analysis determined hub genes and 6 molecular complexes. Functional enrichment analysis revealed that immunity, metabolism, and inflammation are involved in the development of STEMI. Besides, 103 related miRNAs were predicted, hsa-miR-124, hsa-miR-130a/b, and hsa-miR-301a/b regulate the largest number of genes; meanwhile, numerous transcription factors were investigated, EVI1, AML1, GATA1, and PPARG are enriched by most genes. At last, ROC curves indicate MS4A3, KLRC4, KLRD1, AQP9, and CD14 own high sensitivity and specificity in the prediction of STEMI.

Further, GO and KEGG analysis have revealed that immunity, metabolism, and inflammation are involved in the mechanism of STEMI development. As is showcased above, numerous enrichment terms were involved in immunity and inflammation, including cell activation involved in immune response, leukocyte activation involved in immune response, negative regulation of the immune system process, regulation of natural killer cell-mediated immunity, and so on. As the keepers of the immune system, Leukocytes possess bidirectional regulation to the development of STEMI; some leukocytes are atherogenic, whereas others are atheroprotective; some sustain inflammation after myocardial infarction while others resolve it.^19^A review has built a blueprint of the therapy strategies of STEMI, previous experimental studies have revealed complex mechanisms regarding the development, reparative, and remodeling of STEMI and modulating inflammation individually based on the characteristics of the patient’s condition in the will benefit patients with STEMI.^20^Similar results could be found in the functional analysis of the potential molecular complex; all 3 potential molecular complexes were involved in the immunity. As a supplement to GO and KEGG analysis, GSEA analysis verified the results of the functional analysis and provided more evidence of metabolism. In addition to the enrichment analysis results suggesting that DEGs are related to cholesterol metabolism, the results of GSEA raised that numerous terms involved in the regulation of cholesterol metabolism, including plasma lipoprotein assembly remodeling and clearance, plasma lipoprotein clearance, LDL clearance, and heparan sulfate/heparin metabolism, which were consistent with current cognition. Besides, the results of GSEA analysis indicated that the regulation of platelet aggregation plug formation was different between SCAD and STEMI, emphasizing that coagulation is also pivotal to the development of STEMI.^21^Moreover, GSEA highlighted inflammation, oxidative stress, and drug metabolism, which could be confirmed in the existing research.^22 - 24^

MiRNAs play key roles in the genesis and progression of STEMI; after screening, hsa-miR-124, hsa-miR-130a/b, hsa-miR-301a/b, hsa-miR-3666, hsa-miR-4295, and hsa-miR-454 were identified as the main enriched miRNAs, numerous studies illustrate that hsa-miR-124 regulates oxidative stress and hypoxia in the development of MI, and could be a potential biomarker as well as the therapeutic target for STEMI.^25 , 26^MiR-130 family, including miR-130a and miR-130b, an analysis shows miR-130 aggravates STEMI by targeting PPAR-γ pathway.^27^Research is needed to explore and validate the connection between hsa-miR-301a/b, hsa-miR-3666, hsa-miR-4295, and hsa-miR-454 and MI, besides miRNAs supported by experiments involved in the development of MI, such as miR-19, miR-23, and others, could be found in the results of prediction.^28 - 30^The results of transcription factor prediction filtered 4 transcription factors, including EVI1, AML1, GATA1, and PPARG. EVI1, Histone-lysine N-methyltransferase MECOM, are involved in the progress of immunity, metabolism, and inflammation.^31 , 32^Similarly, AML1, runt-related transcription factor 1, is involved in the functional regulation of leukemia, B-cell, and T-cell and regulates the immunity system.^33^GATA1, also known as the Erythroid transcription factor, is involved in the progress of platelet production and coagulation.^34^Meanwhile, a study has elucidated that GATA1 is related to a familial vascular disease with features of SCAD and STEMI.^35^PPARG, known as Peroxisome proliferator-activated receptor gamma, is the nuclear receptor that binds peroxisome proliferators such as hypolipidemic drugs and fatty acids, mainly involved in the progress of fat metabolism and inflammation and is pivotal to the development of STEMI.^36^

MS4A3 regulates the level of phosphorylation of CDK2 through its direct binding to CDKN3,^37^and a cohort study suggests that CDK2 was involved in abnormal proliferation, one of the characteristics of atherosclerosis and STEMI.^38^Besides, a study has mentioned that CDK2 is involved in the regulation of the cell cycle in myocytes after myocardial infarction, which promotes the regeneration of muscle mass and the recovery of ventricular function.^39^Both KLRD1 and KLRC4 are natural killer cell receptors, and natural killer cells are important in the onset of STEMI by their ability to secrete IFN-γ and other inflammatory cytokines.^40^Researchers have mentioned that the overexpression of miR-212 inhibited AQP9 by activating the PI3K/Akt signaling pathway, thus decreasing cardiomyocyte apoptosis, promoting vascular regeneration, and alleviating ventricular remodeling in rats with STEMI.^41^Similarly, a study indicated that silencing the AQP9 gene can inhibit the activation of the ERK1/2 signaling pathway, attenuate the inflammatory response in rats with STEMI, inhibit apoptosis of myocardial cells, and improve cardiac function.^42^CD14, full protein name is monocyte differentiation antigen CD14, recently, a study has mentioned that compared with CAD patients, the CD14-related monocyte levels were significantly higher in patients with STEMI.^43^

This study has some limitations. First, all the results of the analysis were derived from previous data sets. Despite the efforts we have made in quality control, the authenticity of the results still needs verification. Moreover, limited by the information contained in GSE59867 and GSE62646, we cannot compare the diagnostic performance of the identified biomarkers with troponin I and T nor evaluate differences in their temporal kinetics. Third, all data we used came from peripheral blood mononuclear cells, not from coronary artery or heart tissue, because it is relatively difficult to obtain coronary artery and heart tissue clinically. Fortunately, previous studies have shown that peripheral blood data also have good reliability.^44 , 45^Finally, although numerous studies supported the potentiality of the potential biomarkers predicted in this study, the results of ROC curves failed to find a gene with high confidence (AUC > 90%), and considerable trials are needed to validate the sensitivity and specificity of potential biomarkers. Nevertheless, this study determined the potential biomarkers and investigated the complex mechanisms of STEMI developed from SCAD, which has promoted the designation of our next plan to explore the mechanisms in the clinical trial soon.

## Conclusion

We revealed that immunity, metabolism, and inflammation are involved in the development of STEMI derived from SCAD, and 5 genes, including MS4A3, KLRC4, KLRD1, AQP9, and CD14, could be employed as candidate biomarkers for STEMI.
